# The effects of exposure to HIV in neonates at a referral hospital in South Africa

**DOI:** 10.1186/s12887-021-02969-6

**Published:** 2021-11-03

**Authors:** Helena Mellqvist, Robin T. Saggers, Anders Elfvin, Elisabet Hentz, Daynia E. Ballot

**Affiliations:** 1 Futurum, County Hospital Ryhov, Jonkoping, Sweden; 2 Department of Pediatrics, Institute of Clinical Sciences, Sahlgrenska Academy, University of Gothenburg, Gothenburg, Sweden; 3 School of Clinical Medicine, Faculty of Health Sciences, University of the Witwatersrand, Johannesburg, South Africa; 4 Department of Paediatrics and Child Health, Charlotte Maxeke Johannesburg Academic Hospital, Jubilee Road, Parktown, Johannesburg, South Africa; 5 Region Västra Götaland, Department of Pediatrics, The Queen Silvia Children’s Hospital, Sahlgrenska University Hospital, Gothenburg, Sweden

**Keywords:** HIV-exposed neonate, HIV-exposed uninfected, HIV-unexposed neonate, HIV positive mother, Neonatal mortality, Neonatal morbidity, Low and middle-income countries; South Africa

## Abstract

**Background:**

Fewer infants are infected with HIV through mother-to-child transmission, making HIV-exposed but uninfected (HEU) infants a growing population. HIV-exposure seems to affect immunology, early growth and development, and is associated with higher morbidity and mortality rates. Currently, there is a lack of information regarding the clinical effects of HIV-exposure during the neonatal period.

**Objectives:**

To identify a possible difference in mortality and common neonatal morbidities in HEU neonates compared to HIV-unexposed neonates.

**Methods:**

This was a retrospective, descriptive study of all neonates admitted to the neonatal unit at Charlotte Maxeke Johannesburg Academic Hospital between 1 January 2017 and 31 December 2018. HEU neonates were compared to HIV-unexposed neonates.

**Results:**

There were 3236 neonates included, where 855 neonates were HEU. The HEU neonates had significantly lower birth weight and gestational age. The HEU neonates had higher rates of neonatal sepsis (19.8% vs 14.2%, OR 1.49, *p* <  0.001), specifically for late onset sepsis, and required more respiratory support. NCPAP and invasive ventilation was more common in the HEU group (36.3% vs 31.3% required NCPAP, *p* = 0.008, and 20.1% vs 15,0% required invasive ventilation, *p* <  0.001). Chronic lung disease was more common among HIV-exposed neonates (12.2% vs 8.7%, OR 1.46, *p* = 0.003). The difference in mortality rates between the study groups was not significant (10.8% of HEU neonates and 13.3% of HIV-unexposed).

**Conclusions:**

HEU neonates had higher rates of neonatal sepsis, particularly late-onset sepsis, required more respiratory support and had higher rates of chronic lung disease. Mortality of HEU neonates was not different HIV-unexposed neonates.

## Background

Human Immunodeficiency Virus (HIV) is one of the world’s biggest health threats. In 2018, the Republic of South Africa had the third highest prevalence of HIV in the world, with 20.4% of adults infected according to the Joint United Nations Programme on HIV/AIDS (UNAIDS) estimations, which equals 7.5 million people. [1] In addition, 260,000 children between the ages of 0–14 years of age were infected. One of the biggest tragedies of the epidemic, is the transmission of the virus from mother to child, which can occur in utero, intrapartum or postnatally (predominantly through breastfeeding). [2, 3] HIV-infection during infancy has a high risk of mortality, with a net survival of 52% at 1 year if infected perinatally and 78% at 1 year post infection if infected through breastfeeding. [4]

In an effort to stop the HIV/AIDS-epidemic, South Africa has implemented the largest treatment programme in the world, with nearly 4.8 million people on treatment. Treatment for prevention of mother-to-child transmission (PMTCT) was given to 87% of HIV-positive, pregnant women in South Africa during 2018. [1] The nation has adapted the Option B+ by World Health Organisation, which is that all pregnant and breastfeeding women should receive lifelong anti-retroviral therapy (ART), regardless of CD4 count or clinical stage. [5] The therapy provided is a fixed-dose combination (FDC) of Tenofovir, Lamivudine or Emtricitabine and Efavirenz. This FDC-therapy should be initiated at the first antenatal care visit, or at least 12 weeks prior to labour. If the mother is consistently on ART, her HIV-exposed infant will receive HIV-prophylaxis of Nevirapine immediately after birth. The infant will be tested for HIV using polymerase chain reaction (PCR) at birth as well as at 10 weeks of age and 6 weeks after cessation of breastfeeding, as early infant diagnosis and treatment has shown to reduce mortality and prevent disease progression [6, 7]. An uninfected infant is given HIV-prophylaxis with Nevirapine daily until at least 6 weeks of age. The mother will then continue with lifelong anti-retroviral therapy. [8] Annually, approximately 53,000 HIV-infections in infants are averted due to PMTCT in South Africa. Still, 4.89% of children with HIV-positive mothers born during 2018 in South Africa, were infected with the virus. [1] The averted infections result in a growing population of HIV-exposed uninfected (HEU) infants.

Several studies have been conducted to specify the health effects of HIV-exposure during early life, with many showing heterogenous results. [9] Some studies have shown increased mortality in HEU children compared to unexposed children [10–12], while others have shown no difference in mortality rates [13, 14]. Another example of this heterogenicity is seen when observing neonatal sepsis, where one large meta-analysis showed that HEU neonates were more than twice as likely to have neonatal Group B Streptococcus (GBS) disease compared to HIV-unexposed neonates. Specifically, no significant difference was found in the rate of early-onset disease but HEU’s were 4.43 times more likely to have late-onset neonatal GBS disease (95% CI: 1.81–10.85; *p* = 0.001). [15] At the same time, a South African study from 2012 showed HEU neonates to have a lower risk of early-onset sepsis and a similar rate of late-onset sepsis compared to HIV-unexposed neonates. [16]

Other research has shown more definitive results. In the HEU population, infectious diseases, such as tuberculosis, pneumonia and lower respiratory tract infection are more frequent and occasionally also more severe; for example, HEU infants with pneumonia have higher rates of treatment failure and in-hospital mortality. [17–20] The disease-causing pathogens amongst HEU children with pneumonia has also been shown to be unusually diverse, with infections caused by *Pseudomonas aeruginosa, Escherichia coli, Pneumocystis jirovecii* and *Aspergillus* spp. [21]

There are several possible reasons for increased mortality and morbidity in HIV exposed infants: differences in environmental factors, including socioeconomic factors, co-infections and ART-exposure, lower birth weight and gestational age and altered immune factors. [3] HIV-exposure is associated with low birth weight (defined as birth weight < 2500 g) and prematurity (defined as gestational age < 37 weeks), and higher proportions of infants are small for gestational age (defined as birth weight for gestational age below the tenth percentile of a standard population). [22, 23] The exposure to a chronic immune activation that HIV causes in the mother has also been shown to affect the infant’s immunology, with lower absolute levels of CD4+ T cells and naive CD8+ T cells, but increased levels of immature double-negative (CD4–CD8–) T cells, which may indicate disturbed thymic function. [24] In addition, HEU infant have lower levels of maternal transferred antibodies at birth. [25]

Charlotte Maxeke Johannesburg Academic Hospital (CMJAH) has developed the Project to Improve Neonatal Care (PRINCE), an on-going clinical audit in order to provide real-time data to identify areas for improvement. The prevalence of HIV amongst antenatal women in the city of Johannesburg in 2015 was 29.6%, in relation to the national point estimate of HIV-prevalence among antenatal women of 30.8%. [26] With the growing population of HEU neonates, more studies are needed investigating this group of patients in order to improve their care.

## Methods

### Aim

The aim of the study was to identify a possible difference in the mortality and the characteristics of the hospital stay in HIV-exposed uninfected neonates compared to HIV-unexposed neonates.

### Study design

This was a secondary analysis of an existing database of neonates admitted to the neonatal unit at CMJAH between 1 January 2017 and 31 December 2018. The inclusion criterion was known maternal HIV-status. Neonates with a birth weight below 500 g were considered non-viable and excluded. Neonates with positive or intermediate birth HIV-PCR were excluded, as well as neonates on whom no HIV-PCR was conducted.

### Definitions

Maternal and neonatal information was collected. Clinical outcome included duration of hospital stay and outcome (death or survival to discharge.) Place of birth was either ‘inborn’ if born at the study hospital or ‘outborn’. Resuscitation in delivery room was defined as the use of face mask ventilation. Diseases were defined according to the Vermont-Oxford Network (VON) definitions. [27] Grades 2 and 3 necrotizing enterocolitis (NEC), diagnosed both clinically and radiologically according to the modified Bell’s staging criteria, were included in the NEC variable. Retinopathy of prematurity included grades 3 and 4, whereas intraventricular haemorrhage included grade 3 and 4. Chronic lung disease was defined as requiring oxygen on day 28 of life. Sepsis was classified as culture proven only, with onset within 72 h of birth being classified as early-onset and after 72 h as late-onset sepsis.

### Database

The neonatal records at CMJAH are stored on the REDCap (Research Electronic Data Capture) database, hosted by University of Witwatersrand. [28] REDCap is a secure web-based programme that aids data capture for the purposes of clinical audit and quality improvement. Every week clinical staff collected data from records of patients discharged from the unit. This information was verified and entered onto the REDCap database. Maternal and infant characteristics, as well as information regarding clinical outcome and hospital stay was retrieved from the database.

### Statistical analysis

Data was analysed using IBM SPSS (Version 26). We described normally distributed continuous variables using mean and standard deviation and categorical variables using frequency and percentages. Skewed data was described using median and interquartile range. We divided the study sample into HIV-exposed uninfected and HIV-unexposed groups. Continuous variables were compared using unpaired t-test or Mann-Whitney U test as appropriate, while categorical variables were compared using the Chi-square test. A 2-sided *p*-value < 0.05 was considered significant.

### Ethical considerations

All methods were carried out in accordance with relevant guidelines and regulations. Since this was a retrospective review of an existing database, informed consent was waived, and ethics approval obtained from the Human Research Ethics Committee (HREC) of the University of the Witwatersrand (clearance certificate number M190873).

## Results

**Fig. 1:**
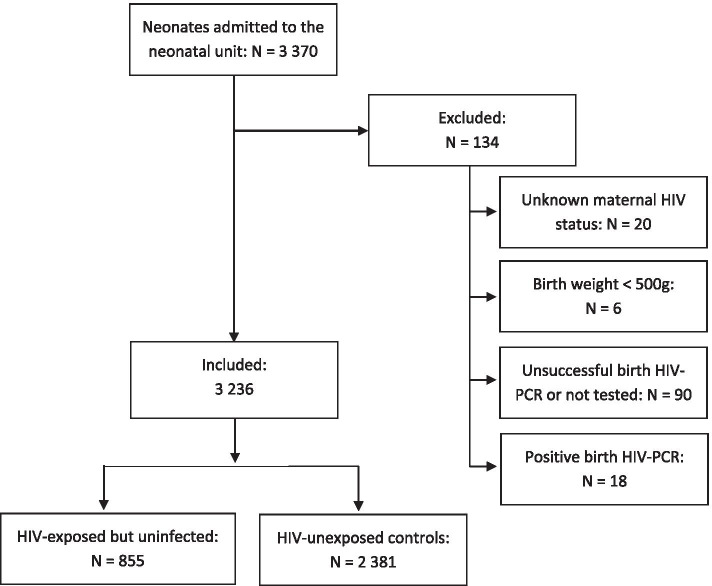
Flowchart of the study population of HIV-exposed and HIV-unexposed neonates

A total of 3370 neonates were admitted to the neonatal unit between 1 January 2017 and 31 December 2018. The inclusion process is described in Fig. 1. Of 3350 neonates with known maternal HIV-status, 962 neonates were born to mothers with HIV and consequently were HIV-exposed (28.7%). Of the HIV-exposed neonates, 879/895 (98.2%) received HIV-prophylaxis and 873 (90.7%) had successful HIV-PCR done at birth. Eighteen of these neonates tested HIV-positive, which resulted in a transmission rate of 2.1% of the tested neonates. Therefore, 855 neonates can be considered as HIV-exposed but uninfected (HEU) at birth and were included in the analysis. The control group consisted of 2381 HIV-unexposed neonates.

The maternal characteristics of the study population are described in Table 1. In summary, the HIV-positive mothers had on average higher age and more pregnancies, but fewer parities (all significant differences with *p*-values < 0.001). The attendance at antenatal care was lower in the HIV-positive group. Other infectious diseases such as tuberculosis and syphilis were more common amongst HIV-positive mothers. The majority of HIV positive mothers (98.5% 801/815) were on ART.

**Table 1:** Maternal characteristics of the study population, separated by maternal HIV-status

Maternal Characteristics	Maternal HIV-status: Positive	Maternal HIV-status: Negative	All mothers	*P*-value
**Maternal age (years)**				
Mean (SD)	30.56 (5.79)	28.35 (6.53)	28.92 (6.41)	< 0.001
Unknown N (%)	105 (12.3%)	262 (11.0%)	367 (11.3%)	
**Parity**				
Median (IQR)	2 (1)	1 (2)	1 (2)	< 0.001
Unknown N (%)	150 (17.5%)	352 (14.8%)	502 (15.5%)	
**Gravidity**				
Median (IQR)	3 (1)	2 (2)	2 (2)	< 0.001
Unknown N (%)	152 (17.8%)	354 (14.9%)	506 (15.6%)	
**Antenatal care**				
N (%)	649 (83.9%)	1979 (87.8%)	2628 (86.8%)	0.006
Unknown N (%)	81 (9.5%)	126 (5.3%)	207 (6.8%)	
**Maternal tuberculosis**				
N (%)	15 (1.9%)	4 (0.2%)	19 (0.6%)	< 0.001
Unknown N (%)	71 (8.3%)	135 (5.7%)	206 (6.8%)	
**Maternal Syphilis**				
N (%)	33 (4.2%)	42 (1.8%)	75 (2.4%)	< 0.001
Unknown N (%)	66 (7.7%)	100 (4.2%)	166 (5.4%)	
**Total**				
N (%)	*855 (26.4%)*	*2381 (71.2%)*	*3236 (100%)*	

*IQR* interquartile range; *SD* standard deviation

The majority of the neonates in both groups were delivered at the study hospital, but more HEU neonates were outborn (203/847, 24.0% compared to 435/2358, 18.4%; *p* <  0.001). HEU neonates also had higher occurrence of vaginal delivery than HIV-unexposed (395/817, 48.3% vs 947/2313, 40.9%; *p* <  0.001). Caesarean section occurred in a total of 1788 cases, with 1459 (82.2%) being emergency surgeries. There was no significant association with HIV-exposure and emergency or elective caesarean section. The characteristics of the included neonates are described in Table 2. The HEU neonates had significantly lower birth weight and gestational age.

**Table 2:** Neonatal characteristics of the study population, separated by HIV-exposure

Neonatal characteristics	HEU	HIV-unexposed	All neonates	*P*-value
**Birth weight (g)**				< 0.001
Mean (SD)	2102 (882)	2265 (995)	2222 (969)	
Unknown N (%)	1 (0.1%)	3 (0.1%)	4 (0.1%)	
**Head circumference (cm)**				0.004
Mean (SD)	31.35 (3.52)	31.81 (3.91)	31.70 (3.82)	
Unknown N (%)	122 (14.3%)	291 (12.2%)	414 (12.8%)	
**Gestational age (weeks)**				0.004
Mean (SD)	33.95 (4.55)	34.49 (4.70)	34.34 (4.67)	
Unknown N (%)	12 (1.4%)	31 (1.3%)	43 (1.3%)	
**Gender = Male**				
N (%) Unknown N (%)	452 (53.0%) 2 (0.2%)	1245 (52.4%) 4 (0.2%)	1697 (52.5%) 6 (0.2%)	0.759
Total				
N (%)	*855 (26.4%)*	*2381 (71.2%)*	*3236 (100%)*	

*HEU* HIV-exposed uninfected; *SD* standard deviation

A total of 408 deaths occurred during the study period: 92/855 (10.8%) were HEU compared to 13.3% (316/2381) of the HIV-unexposed neonates. The difference in mortality between the study groups was not significant (*p* = 0.058). The median age on outcome (discharge or death) was 6 days in the HIV-unexposed group and 8 days in the HEU group (*p* = 0.005). The median length of stay at the neonatal unit was 6 days in the HIV-unexposed group and seven in the HEU group (*p* = 0.015). When defined as early neonatal mortality (death within the first 7 days of life) to take days at risk into consideration, the difference in mortality was still not significant.

**Fig. 2:**
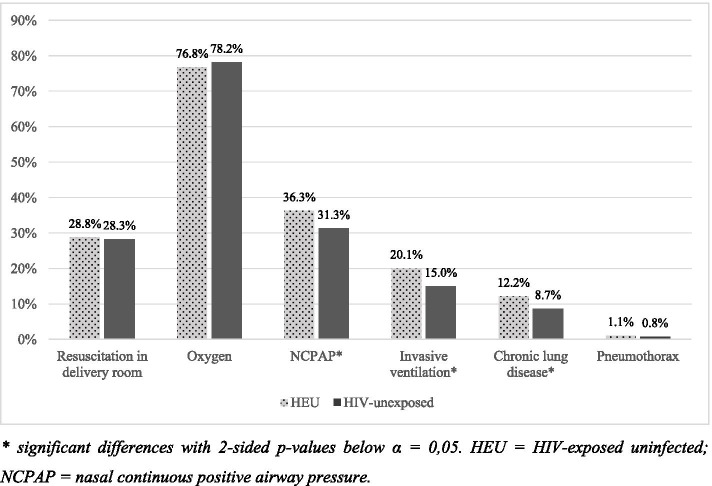
Use of respiratory support and respiratory morbidities between HIV-exposed uninfected and HIV-unexposed neonates. * significant differences with 2-sided *p*-values below α = 0,05. HEU = HIV-exposed uninfected; NCPAP = nasal continuous positive airway pressure

The difference in respiratory support and diagnoses are shown in Fig. 2. Initial resuscitation in the delivery room and oxygen after initial resuscitation was required equally in both groups, but HIV-exposed neonates had higher frequency of respiratory support requirements after initial resuscitation. NCPAP was required by 36.3% of HEU, compared to 31.3% of HIV-unexposed (*p* = 0.008). Invasive ventilation was also required more by HEU (*p* < 0.001), yet with no significant difference in the duration of respiratory support between the study groups.

Chronic lung disease was more common amongst HEU neonates (*p* = 0.003) with an OR of 1.46 (95% CI 1.13–1.88). Twenty-eight neonates had pneumothorax (nine HEU and 19 HIV-unexposed), with no significant difference in frequency between the study groups.

A total of 506 neonates had neonatal sepsis during the time-period studied. Thirty-eight of these neonates had both early- and late-onset sepsis. The distribution between the HIV-exposure groups is shown in Table 3. Any neonatal sepsis (early and/or late) was associated with HIV-exposure. LOS was significantly more common amongst HEU (16.5% compared to 12.1%), but EOS did not have a significant association with HIV-exposure on its own.

**Table 3:** Comparison of early-onset sepsis, late-onset sepsis, or any neonatal sepsis among neonates, separated by HIV-exposure

	HEU	HIV-unexposed	OR (95% CI)	*P*-value
EOS: N (%)	36 (4.2%)	79 (3.4%)		0.210
LOS: N (%)	141 (16.5%)	288 (12.1%)	1.44 (1.15–1.79)	0.001
Any neonatal sepsis: N (%)	169 (19.8%)	337 (14.2%)	1.49 (1.22–1.80)	< 0.001

*EOS* early-onset sepsis; *HEU* HIV-exposed uninfected; *LOS* late-onset sepsis; *OR* odds ratio

Other neonatal diseases such as necrotising enterocolitis, retinopathy of prematurity, intraventricular haemorrhage and patent ductus arteriosus were not significantly different between the two groups. Clinical features at birth such as hypothermia and low 5-min Apgar score was also not significantly different between the two groups.

## Discussion

This study found an association between HIV exposure and higher rates of neonatal sepsis, specifically LOS, and the requirement of more respiratory support. The characteristics of the included neonates and mothers in this study are potentially not representable for all babies born at the study site, as the study population only included neonates admitted to the neonatal unit. Realistically, the mean birth weight and gestational age in the study group are lower in the study population than in the general population of neonates born at the study site, including full-term, healthy neonates. HIV-infected children have more hospital admissions, making it possible for the HIV-exposure rate to be falsely high. [11] This argument is also strengthened by a study from the paediatric intensive care unit at CMJAH from 2013 to 2014 which showed a HIV-exposure rate of 34.1%, potentially caused by more severe illnesses amongst HIV-exposed children. [29] Nationally in 2017, 28.7% of women receiving antenatal care were HIV-positive, but in another urban hospital in Johannesburg the rate was lower at 23.2%. [30, 31]

A study conducted at the neonatal unit at CMJAH between 2015 and 2017 (overlapping the study period of this report, and therefore including some of the same population) showed comparable HIV-exposure rate of 28.6%, infection rate of 2.52% and a birth HIV-PCR rate of 88.1%. [32] Notable is a trend towards lower infection rate and higher rates of PCR done at birth in this study, but since the years are not separated in either of the studies a statistical comparison cannot be made fully. The 90.7% birth HIV-PCR rate found in this study shows great potential for improvement as ideally it should be 100%. Neonates with indeterminate or invalid PCR result contribute to lowering this rate. With the current laboratory testing system, there is a lag of approximately three days between taking a PCR test and obtaining the result. As point-of-care testing becomes more widely accessible, birth HIV-PCR results may become more readily available.

The findings of this study also strengthen previous reports on HIV-exposure’s association with birth at lower gestational age and lower birth weight. The reason as to why this difference exists is not yet established. Many have discussed that the main factor is unhealthier mothers, not only from the chronic disease but also since HIV-infected women in South Africa often have lower socioeconomic status. In a large meta-analysis, HIV-exposed neonates were more likely to have low birth weight in developing countries compared with women in developed countries showing maternal health to be a strong contributing factor. [22] Keeping this in mind, this association with low birth weight was demonstrated in developed countries, although not as strongly linked, making the association not fully explained.

The mortality rates in this study were not significantly different between the study groups, despite the differences in characteristics and illness. The increased requirement of respiratory support was also found in the study from 2013 to 2014; HIV exposure may be associated with more severe illness. [29] Another possible reason is more prematurity amongst HIV-exposed neonates. Interestingly, neonatal sepsis and specifically LOS was found to be significantly more common amongst HEU neonates, which previously had shown heterogenous associations. Other neonatal morbidities were not found to be significant.

Possible confounders in this study are maternal factors other than HIV-infection, such as comorbidities like maternal tuberculosis and/or syphilis and higher maternal age. Additionally, HIV-positive women were found to have more pregnancies and lower number of parities. A possible reason for this is due to more miscarriages, as other studies have shown, but that information was not collected in this study. [33] This finding could be a possible new field of research.

A question remaining is the causative relationship, as HIV-exposure is not only exposure to the virus and chronic maternal immune activation – it is also exposure to ART-drugs, vertical transmission of other pathogens and poorer maternal health. Other studies have shown that these environmental factors cannot explain the association fully, since HIV-exposure seems to affect neonatal immunology. [24, 25] Further studies establishing the causes are needed in order to identify preventable reasons and to bridge the health gap between HIV-exposed and unexposed neonates.

### Methodological considerations

As this is a retrospective study, there are some methodological aspects to consider. The analysis was limited by unavailable information, where maternal disease status, CD4 count, duration of ART, and high or low risk of HIV-transmission was unknown. The study also did not take multiple births into account. The benefit of the chosen method is that a large study population was available for analysis.

The aim of the study was to compare the rates of mortality and morbidity between HEU and HU neonates, only associations are described rather than causative relationships. Since the study was based on an admitted population, there are limitations as to the applicability of the results. These results are not applicable to all neonates but may be applicable to other admitted populations in similar settings.

## Conclusions

Just less than a third (28,7%) of neonates admitted during the study period were HIV-exposed uninfected. This study suggests that HIV-exposure amongst neonates is associated with birth at a lower gestational age and lower birth weight. HIV-exposure was also associated with increased rates of neonatal sepsis, respiratory support, and chronic lung disease. The clinical consequences that should follow affects the care of both mothers and neonate. HIV-infected women might benefit from more nutritional education and support during pregnancy in antenatal care facilities, to help close the gap in birth weight and gestational age. Health care providers caring for HIV-exposed neonates should be mindful of the increased risk in neonatal sepsis and respiratory disease, in order to identify and if possible, treat these severe illnesses at an early stage.

## Data Availability

The data that support the findings of this study are available on request from the corresponding author [RTS]. The data are not publicly available due to them containing information that could compromise research participant privacy.

## Abbreviations

AIDS: Acquired Immunodeficiency Syndrome
ART: Antiretroviral therapy
CI: Confidence interval
CMJAH: Charlotte Maxeke Johannesburg Academic Hospital
EOS: Early-onset sepsis
FDC: Fixed dose combination
GBS: Group B Streptococcus
HEU: HIV-exposed uninfected
HIV: Human Immunodeficiency Virus
IQR: Interquartile range
LOS: Late-onset sepsis
NEC: Necrotizing enterocolitis
NCPAP: Nasal continuous positive airway pressure
PCR: Polymerase chain reaction
PMTCT: Prevention of mother to child transmission
REDCap: Research Electronic Data Capture
SD: Standard deviation
VON: Vermont Oxford Network

## References

[1] (UNAIDS) UNPoHA. AIDS Info [2019-09-11]. Country Factsheet, South Africa - 2018]. Available from: http://aidsinfo.unaids.org/?did=5b4e7dc0dddb54192bb396e4&r=world&t=null&tb=q&bt=undefined&ts=0,0&qla=C&qls=ZAF.

[2] Kourtis AP, Bulterys M (2010). Mother-to-child transmission of HIV: pathogenesis, mechanisms and pathways. Clin Perinatol.

[3] Auriti C, De Rose DU, Santisi A, Martini L, Piersigilli F, Bersani I (1867). Pregnancy and viral infections: mechanisms of fetal damage, diagnosis and prevention of neonatal adverse outcomes from cytomegalovirus to SARS-CoV-2 and Zika virus. Biochim Biophys Acta Mol basis Dis.

[4] Marston M, Becquet R, Zaba B, Moulton LH, Gray G, Coovadia H (2011). Net survival of perinatally and postnatally HIV-infected children: a pooled analysis of individual data from sub-Saharan Africa. Int J Epidemiol.

[5] (WHO) WHO. Guideline on when to start antiretroviral therapy and on pre-exposure prophylaxis for HIV. 2015. 26598776

[6] National department of health SA. National consolidated guidelines for the prevention och mother-to-child transmission of HIV (PMTCT) and the management of HIV in children, adolescents and adults. In: Department of Health SA, editor. www.doh.gov.za2015.

[7] Violari A, Cotton MF, Gibb DM, Babiker AG, Steyn J, Madhi SA (2008). Early antiretroviral therapy and mortality among HIV-infected infants. N Engl J Med.

[8] Provincial Government of the Western Cape- Department of Health HASTHD. The Western Cape Consolidated Guidelines for HIV Treatment: Prevention of Mother-to- Child Transmission of HIV (PMTCT), Children, Adolescents and Adults. 2018.

[9] Afran L, Garcia Knight M, Nduati E, Urban BC, Heyderman RS, Rowland-Jones SL (2014). HIV-exposed uninfected children: a growing population with a vulnerable immune system?. Clin Exp Immunol.

[10] Chilongozi D, Wang L, Brown L, Taha T, Valentine M, Emel L (2008). Morbidity and mortality among a cohort of human immunodeficiency virus type 1-infected and uninfected pregnant women and their infants from Malawi, Zambia, and Tanzania. Pediatr Infect Dis J.

[11] Shapiro RL, Lockman S, Kim S, Smeaton L, Rahkola JT, Thior I (2007). Infant morbidity, mortality, and breast milk immunologic profiles among breast-feeding HIV-infected and HIV-uninfected women in Botswana. J Infect Dis.

[12] Ruperez M, Gonzalez R, Maculuve S, Quinto L, Lopez-Varela E, Augusto O (2017). Maternal HIV infection is an important health determinant in non-HIV-infected infants. Aids..

[13] Rollins NC, Ndirangu J, Bland RM, Coutsoudis A, Coovadia HM, Newell ML (2013). Exclusive breastfeeding, diarrhoeal morbidity and all-cause mortality in infants of HIV-infected and HIV uninfected mothers: an intervention cohort study in KwaZulu Natal, South Africa. PLoS One.

[14] Chopra M, Doherty T, Goga A, Jackson D, Persson LA (2010). Survival of infants in the context of prevention of mother to child HIV transmission in South Africa. Acta Paediatr.

[15] Cools P, van de Wijgert J, Jespers V, Crucitti T, Sanders EJ, Verstraelen H (2017). Role of HIV exposure and infection in relation to neonatal GBS disease and rectovaginal GBS carriage: a systematic review and meta-analysis. Sci Rep.

[16] Cutland CL, Schrag SJ, Zell ER, Kuwanda L, Buchmann E, Velaphi SC (2012). Maternal HIV infection and vertical transmission of pathogenic bacteria. Pediatrics..

[17] Marquez C, Chamie G, Achan J, Luetkemeyer AF, Kyohere M, Okiring J (2016). Tuberculosis infection in early childhood and the association with HIV-exposure in HIV-uninfected children in rural Uganda. Pediatr Infect Dis J.

[18] Koyanagi A, Humphrey JH, Ntozini R, Nathoo K, Moulton LH, Iliff P (2011). Morbidity among human immunodeficiency virus-exposed but uninfected, human immunodeficiency virus-infected, and human immunodeficiency virus-unexposed infants in Zimbabwe before availability of highly active antiretroviral therapy. Pediatr Infect Dis J.

[19] Cohen C, Moyes J, Tempia S, Groome M, Walaza S, Pretorius M (2016). Epidemiology of acute lower respiratory tract infection in HIV-exposed uninfected infants. Pediatrics..

[20] Kelly MS, Wirth KE, Steenhoff AP, Cunningham CK, Arscott-Mills T, Boiditswe SC (2015). Treatment failures and excess mortality among HIV-exposed, uninfected children with pneumonia. J Pediatric Infect Dis Soc.

[21] McNally LM, Jeena PM, Gajee K, Thula SA, Sturm AW, Cassol S (2007). Effect of age, polymicrobial disease, and maternal HIV status on treatment response and cause of severe pneumonia in south African children: a prospective descriptive study. Lancet..

[22] Xiao PL, Zhou YB, Chen Y, Yang MX, Song XX, Shi Y (2015). Association between maternal HIV infection and low birth weight and prematurity: a meta-analysis of cohort studies. BMC Pregnancy Childbirth.

[23] Sania A, Smith ER, Manji K, Duggan C, Masanja H, Kisenge R (2018). Neonatal and Infant Mortality Risk Associated with Preterm and Small for Gestational Age Births in Tanzania: Individual Level Pooled Analysis Using the Intergrowth Standard. J Pediatr.

[24] Clerici M, Saresella M, Colombo F, Fossati S, Sala N, Bricalli D (2000). T-lymphocyte maturation abnormalities in uninfected newborns and children with vertical exposure to HIV. Blood..

[25] de Moraes-Pinto MI, Almeida AC, Kenj G, Filgueiras TE, Tobias W, Santos AM (1996). Placental transfer and maternally acquired neonatal IgG immunity in human immunodeficiency virus infection. J Infect Dis.

[26] Department of Health RoSA. The 2015 National Antenatal Sentinel HIV & Syphilis Survey 2017.

[27] Vermont Oxford Network [Available from: https://public.vtoxford.org/].

[28] Harris PA, Taylor R, Thielke R, Payne J, Gonzalez N, Conde JG (2009). Research electronic data capture (REDCap)--a metadata-driven methodology and workflow process for providing translational research informatics support. J Biomed Inform.

[29] Mopeli RKB, D.E.; White, D.A. (2016). An audit of primary medical conditions in children admitted to the paediatric intensive care unit of Charlotte Maxeke Johannesburg academic hospital. S Afr J Child Health.

[30] Woldesenbet SA, Kufa T, Barron P, Chirombo BC, Cheyip M, Ayalew K, et al. Viral suppression and factors associated with failure to achieve viral suppression among pregnant women in South Africa: a national cross-sectional survey. Aids. 2019. 10.1097/QAD.0000000000002457 PMC7050794 31821189

[31] Technau KG, Kuhn L, Coovadia A, Carmona S, Sherman G (2017). Improving early identification of HIV-infected neonates with birth PCR testing in a large urban hospital in Johannesburg, South Africa: successes and challenges. J Int AIDS Soc.

[32] Benali G, Ramdin T, Ballot D (2019). An audit of mother to child HIV transmission rates and neonatal outcomes at a tertiary hospital in South Africa. BMC Research Notes.

[33] Malaba TR, Phillips T, Le Roux S, Brittain K, Zerbe A, Petro G (2017). Antiretroviral therapy use during pregnancy and adverse birth outcomes in south African women. Int J Epidemiol.

